# Identification and Virulence Characterization of Two *Akanthomyces attenuatus* Isolates Against *Megalurothrips usitatus* (Thysanoptera: Thripidae)

**DOI:** 10.3390/insects10060168

**Published:** 2019-06-13

**Authors:** Cailian Du, Bo Yang, Jianhui Wu, Shaukat Ali

**Affiliations:** Key Laboratory of Bio-Pesticide Innovation and Application, Engineering Research Centre of Biological Control, South China Agricultural University, Guangzhou 510642, China; Ducailian@stu.scau.edu.cn (C.D.); yangb19940629@163.com (B.Y.); jhw@scau.edu.cn (J.W.)

**Keywords:** *Megalurothrips usitatus*, *Akanthomyces attenuates*, entomopathogenic fungi, biological control

## Abstract

*Megalurothrips usitatus* (Bagnall) is one of the most harmful pests of leguminous plants. In order to expand our knowledge on the infection of *M. usitatus* by entomopathogenic fungi, two newly identified isolates of *Akanthomyces attenuatus* (Zare & Gams) were tested for their pathogenicity against *M. usitatus*. Both isolates of *A. attenuatus* (SCAUDCL-38 and SCAUDCL-56) were isolated from soil and were identified by morphological and molecular analyses. The adult females of *M. usitatus* were treated with five different concentrations (1 × 10^4^, 1 × 10^5^, 1 × 10^6^, 1 × 10^7^, and 1 × 10^8^ conidia/mL) of the isolates. Our results revealed 76.25% and 57.5% mortality of *M. usitatus* after five days of treatment with 1 × 10^8^ conidia/mL of SCAUDCL-38 and SCAUDCL-56, respectively. The median lethal concentrations (LC_50_) of SCAUDCL-38 and SCAUDCL-56 calculated through linear regression analysis after five days of fungal treatment of *M. usitatus* were 1.9 × 10^6^ and 1.5 × 10^7^ conidia/mL, respectively, whereas the median lethal time (LT_50_) observed for 1 × 10^8^ conidia/mL of SCAUDCL-38 and SCAUDCL-56 were 3.52 days and 4.9 days, respectively. *A. attenuatus* isolates SCAUDCL-38 and SCAUDCL-56 are highly pathogenic strains of *M. usitatus*. These findings offer valuable information on the development and commercialization of alternative control measures against *M. usitatus*.

## 1. Introduction

*Megalurothrips usitatus* (Bagnall) (Thysanoptera: Thripidae), also known as bean flower thrips, is a major threat to snap bean and cowpea in southern regions of China [1]. Direct damage by thrips reduces the photosynthetic ability of the host plants [2]. Indirect losses due to fruit malformation and scarring caused by thrips are of greater economic significance compared to the direct losses [3,4]. The frequent use of conventional broad-spectrum chemical pesticides has dominated the management of *M. usitatus* [5]. The long-term use of synthetic chemicals to manage the *M. usitatus* is causing environmental pollution and adverse effects to live organisms [6]. This heavy application of pesticides has also resulted in the interruption of the biological balance between natural enemies and insect pests [5,7]. The above-mentioned circumstances have increased the awareness of the necessity as well as the desire to develop pest control strategies that are environmentally safe and biodegradable [8].

Many recent studies have shown that entomopathogenic fungi such as *Metarhizium anisopliae* Sorokin, *Metarhizium brunneum* Petch, *Beauveria bassiana* (Balsamo) Vuillemin, and *Isaria fumosorosea* Wize are effective against different thrips species [9,10,11,12,13,14,15]. *Akanthomyces attenuatus* Zare & Gams (previously known as *Lecanicillium attenuatus*, now designated as belonging to *Akanthomyces* clade, Pong et al. [16]) is a well-known pathogen of whitefly, aphid, and Thrips [17]. Some strains of this species have been developed as commercial biopesticides [17,18]. *A. attenuatus* is pathogenic to a variety of insect orders and mite groups [19,20]. Therefore, *A. attenuatus* may prove to be an effective microbial control option which can suppress *M. usitatus* populations. As the pathogenicity of *A. attenuatus* against *M. usitatus* may vary among different isolates [12], improvement in the basic knowledge as well as increasing the existing pool of *A. attenuatus* isolates can help in the selection of the most suitable isolate for commercial use.

This study presents the isolation, identification, and description of two *A. attenuatus* strains from China. The isolated strains were also tested for their pathogenicity against bean flower thrips, which can provide valuable information for the potential development of *A. attenuatus* as an effective bio-pesticide against *M. usitatus*.

## 2. Materials and Methods

### 2.1. Collection of Soil and Isolation of Fungi

Soil samples were collected from cultivated fields at the South China Agricultural University (SCAU), Guangzhou, China, beneath surface litter (to a depth of 10 cm), were individually placed in polyethylene bags, and were held at −4 °C until they were processed. Fungal isolation was performed following Inglis et al. [21] and Imoulan et al. [22]. Briefly, 3 g of soil was added to 30 mL sterile ddH_2_O containing 0.05% Tween-80. The mixture was stirred for 15 min on a time-controlled magnetic stirrer. After stirring, 1 mL suspension was inoculated to Petri dishes containing potato dextrose agar (PDA), and the plates were incubated at 25 ± 1 °C and 80 ± 5% R.H., with a 16:8 h (Light/ Dark) photoperiod. The Petri dishes were monitored for fungal sporulation after 7 days, which was followed by inoculation of individual germlings on new PDA plates. In this way, several rounds of inoculation were performed until a purified culture, based on phenotypic characteristics and fungal morphology, was obtained [12].

### 2.2. Insect Rearing

The population of *M. usitatus* originated in 2017 from a cowpea field in Guangzhou, China. This population was subsequently reared by the bean pod method. The colony was kept in a growth chamber at 26 ± 6 °C, (70 ± 5)% RH, and 16:8 h (Light/Dark)photoperiod.

### 2.3. Morphological Characterization

The morphological characteristics of two isolates (SCAUDCL-38 and SCAUDCL-53) were observed by culturing a small piece of fungal mycelia on a block of PDA overlaid by a coverslip for 10 days [23]. The slides were stained with lactophenol cotton blue and observed at 40X under a phase-contrast microscope. Conidial images were captured digitally with an Axio Cam HRc camera (Carl Zeiss) using the Axion Vision SE64 Release 4.9.1 software.

### 2.4. Radial Growth and Conidial Yield

The average daily growth rate and conidial yields of both strains were determined using the method of Ali et al. [24]. Fungal mycelial plugs (1 cm diameter) obtained from basic culture (as mentioned in Section 2.1) were cultured on fresh PDA plates for 10 days. The colony diameter was measured on a daily basis. After 10 days of growth, the conidia were scraped from the Petri dishes and suspended in 100 mL of 0.05% Tween-80. The conidial suspension was filtered through muslin cloth to remove the mycelia. The conidial concentration in suspension was quantified using a hemocytometer a phase-contrast microscope at 40X under.

### 2.5. DNA Extraction, PCR Amplification, and Sequence Analysis

The genomic DNA of the purified fungal strains (SCAUDCL-38 and SCAUDCL-56) was extracted with a fungal DNA isolation kit (Ezup, Sangon Biotech, Shanghai, China). The genomic DNA was used as a template for PCR amplification of the internal transcribed spacer (ITS) and elongation factor 1 alpha (TEF or EF1-α) regions [23,25]. All PCR reactions were performed in a 50 μL reaction system, which contained 25 μL 2 × Tap PCR Master (1 μL of each primer (10 μM), 1 μL genomic DNA, and 22 μL ddH_2_O). The ITS regions were amplified using the universal primers reported in Table 1 using the following cycling conditions: 5 min at 94 °C, 35 cycles at 94 °C for 30 s, 53 °C for 30 s, 72 °C for 1 min, and a final extension at 72 °C for 10 min. The EF1-α regions were amplified using the primers in Table 1. Touch-down PCR amplifications were performed under the following conditions: denaturation at 94 °C for 2 min, annealing temperature for the first amplification cycle 66 °C with subsequent reductions of 1 °C per cycle over the next nine cycles. Additional amplification cycles (36 cycles) were performed through denaturation at 94 °C for 30 s, annealing at 56 °C for 30 s, and final incubation at 72 °C for 10 min. The purity of the PCR products was confirmed through agarose gel electrophoresis followed by staining with GenGreen (TianGen Biotech, Beijing, China). The purified PCR products were dispatched to Shanghai Majorbio Bio-pharm Technology (Shanghai, China) for complete bidirectional sequencing with PCR primers.

The sequences were spliced with Genious version 7.1.4 and were blasted using GenBank, followed by connecting two gene fragments in series with TextPad 8.0.2. The sequences were compared using MEGA Version 7.0 [29] and the Kimura-2-parameter (K2P) to calculate a Maximum Likelihood (ML) tree [30].

### 2.6. Virulence of the Fungi in the Laboratory

*Akanthomyces attenuatus* isolates (SCAUDCL-38 and SCAUDCL-56) were cultured for 10 days on PDA plates and suspended in 0.01% Tween-80 as described in Section 2.3 to prepare a suspension containing 1 × 10^8^ conidia/mL. Lower concentrations (1 × 10^7^, 1 × 10^6^, 1 × 10^5^, and 1 × 10^4^ conidia/mL) were prepared through serial dilutions. The pathogenicity of *A. attenuatus* strains (SCAUDCL-38 and SCAUDCL-56) against adult *M. usitatus* under laboratory conditions was studied through a centrifuge tube residual bioassay. The centrifuge tubes along with a soya bean pod (1 cm length) were immersed individually in each conidial concentration for 2 hours. The centrifuge tubes and soya bean pods immersed in 0.01% Tween-80 only served as the control. Healthy females of *M. usitatus* (100 individuals) were transferred to each tube using a camel-hair brush. The tubes were sealed with a cotton plug to prevent thrips from escaping and were incubated in a growth chamber at 26 ± 1 °C, 75% R.H., and 16:8 h (Light/Dark) photoperiod. The insects were observed on a daily basis to record the number of dead *M. usitatus* females. The *M. usitatus* females infected with *A. attenuatus* were identified by the method of Ali et al. [31].

The complete experiment was performed three times using freshly prepared fungal suspensions.

### 2.7. Transmission Electron Microscopy

M. *usitatus* individuals were inoculated with 1.0 × 10^8^ conidia/mL of each strain and incubated at 26 °C and 75% relatively humidity. Gross changes in the appearance of the infected *M. usitatus* were directly monitored at different times after inoculation under a JEM1011 Transmission electron microscope (Nikon Co. Ltd., Japan). The infected *M. usitatus* were sampled at 1, 2, 3, 4, and 5 days after inoculation. They were fixed in 2.5% glutaraldehyde solution and then treated according to the method previously described [32].

### 2.8. Statistical Analysis

Radial growth and conidial yield data were subjected to a one-way ANOVA, and the means were compared using Tukey’s HSD test at a 5% level of significance. Mortality data were percent-transformed and subjected to probit analysis to calculate the medial lethal concentration (LC_50_) and the median lethal time (LT_50_) [33]. All the analyses were performed through SAS 9.1 [34].

## 3. Results

### 3.1. Morphological Identification of Fungi

**Figure 1:**
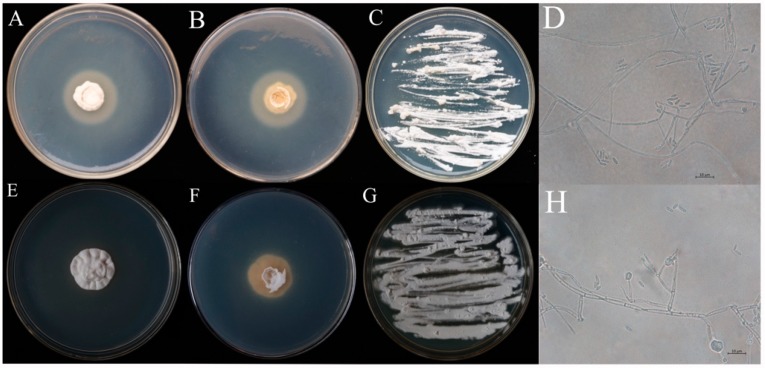
Colony morphology and conidia morphology of two *Akanthomyces attenuatus* isolates (SCAUDCL-38 and SCAUDCL-56). (**A**–**C**) Colony morphology of 10-day-old SCAUDCL-38; (**D**) conidia of isolate SCAUDCL-38; (**E**–**G**) colony morphology of 10-day-old SCAUDCL-56; (**H**) conidia of isolate SCAUDCL-56.

Two strains of entomopathogenic fungi SCAUDCL-38 and SCAUDCL-56 were successfully isolated from soil during this study. Both strains grew well on PDA plates (Figure 1A,B,E,F). The two strains exhibited different morphological characteristics. The morphological characteristics of the two isolates are reported below.

SCAUDCL-38: The mycelia were hyaline or light-colored, with septate branching having the width of 1.2–2.0 μm and 1 or 2 branches. The mycelial joint was thick, while the tip was sharp. The total length was 14.5–23.0 × 0.9–1.6 μm. The conidia were either long or short, with an elliptical shape and were transparent and light (1.5–1.7 × 2.6–6.0 μm) (Figure 1D). The colony diameter after 10 days was 16 mm, and the conidial yield was 5.45 × 10^6^ conidia/mL.

SCAUDCL-56: The mycelia were hyaline or light-colored, with septate branching. The width of the mycelium was 1.4–2.1 μm, with 3–4 whorls. The mycelial joint was thick, while the tip was sharp. The total length was 10–20.7 × 1.1–2.3 μm. The conidia were long or short, had an elliptical shape, and were transparent and light (1.3–2.2 × 3.2–8.2 μm) (Figure 1H). After 10 days, the colony diameter was 22.5 mm, and the spore yield was 1.50 × 10^7^ conidia/mL.

After morphological observation, both strains (SCAUDCL-38 and SCAUDCL-56) were preliminarily identified as *A. attenuatus*.

### 3.2. Molecular Analyses

The purified DNA was amplified by PCR to obtain partial 18s rDNA, ITS, and *EF* 1-α sequences.

#### 3.2.1. BLASTN Comparisons

The comparison of the results in GenBank showed that the ITS sequence of SCAUDCL-38 had 100%, 99.83%, and 99.48% similarity to *Akanthomyces* strain sequences in GenBank (GenBank Accession No.MH558279, LT992877, and MH231313). SCAUDCL-56 had 99.83–100% similarity to *Akanthomyces* strain sequences in GenBank (GenBank Accession No.MH558279, LT992877).The *EF* 1-α sequences of SCAUDCL-38 and SCAUDCL-56 had 98–100% similarity to *Akanthomyces* strains in GenBank. For details of the sequences in GenBank used above, see Table 2.

#### 3.2.2. Phylogenetic Analysis

**Figure 2:**
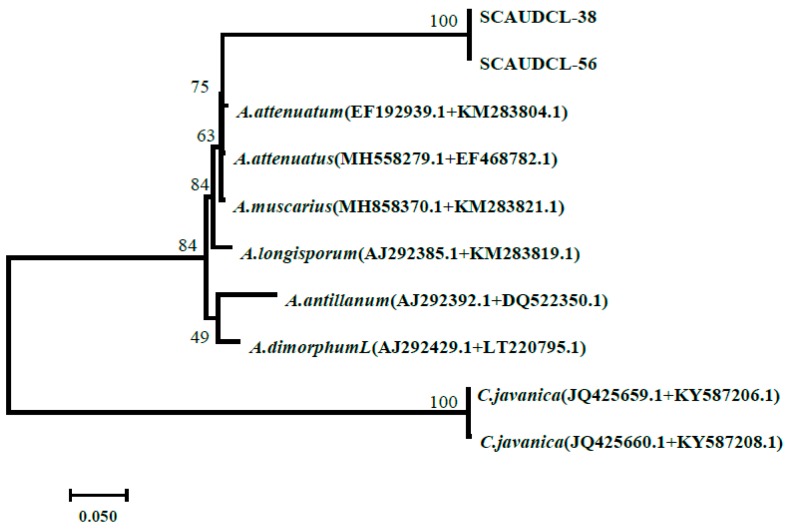
Majority rule consensus phylogram from the Maximum Likelihood (ML) tree based on the sequences of the ITS region and of the protein-coding gene translation elongation factor 1 alpha. (*EF*1-α) for two *A. attenuatus* isolates (SCAUDCL-38 isolate, SCAUDCL-56). *C. javanica* was used as an outgroup.

Our results showed that strains were closely similar to *Akanthomyces spp.* Both SCAUDCL-38 and SCAUDCL-56 clustered together with strains of *A. attenuatus* (GenBank Accession No.EF192939+KM283204) with a bootstrap value of 75% (Figure 2).

### 3.3. Virulence of A. attenuatus against M. usitatus

**Figure 3:**
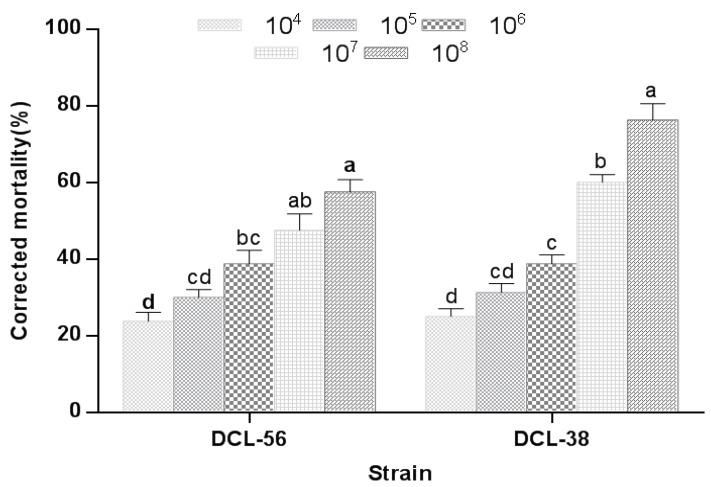
Corrected mortality of *Megalurothrips usitatus* at five days post-inoculation with different conidial concentrations of *A. attenuates* isolates. Different lowercase letters above the bar for each isolate indicate significant differences at the level of *p* < 0.05 determined by Tukey’s HSD test.

Both putative *A. attenuatus* strains (SCAUDCL-38 and SCAUDCL-56) were pathogenic to *M. usitatus.* The pathogenicity of the two strains against *M. usitatus* increased with increasing conidial concentration (Figure 3). *A. attenuatus* strain SCAUDCL-38 was more virulent than SCAUDCL-56 against *M. usitatus*. There was no significant difference in the adjusted mortality of *M. usitatus* caused by SCAUDCL-38 and SCAUDCL-56 when the insects were treated with lower conidial concentrations (1 × 10^4^, 1 × 10^5^ and 1 × 10^6^ conidia/mL). However, at higher concentrations (1 × 10^7^ and 1 × 10^8^ conidia/mL), the strain SCAUDCL-38 (76.25%) induced significantly higher *M. usitatus* mortality than the strain SCAUDCL-56 (57.5%) (Figure 3). LC_50_ values of SCAUDCL-38 and SCAUDCL-56 against *M. usitatus* were 1.9 × 10^6^ and 1.5 × 10^7^ conidia/mL, respectively. The LT_50_ values of SCAUDCL-38and SCAUDCL-56 (when 1 × 10^8^ conidia/mL was applied) were 3.5 and 4.9 days, respectively (Table 3 and Table 4).

### 3.4. Microscopic Examination of A. attenuatus Infection

**Figure 4:**
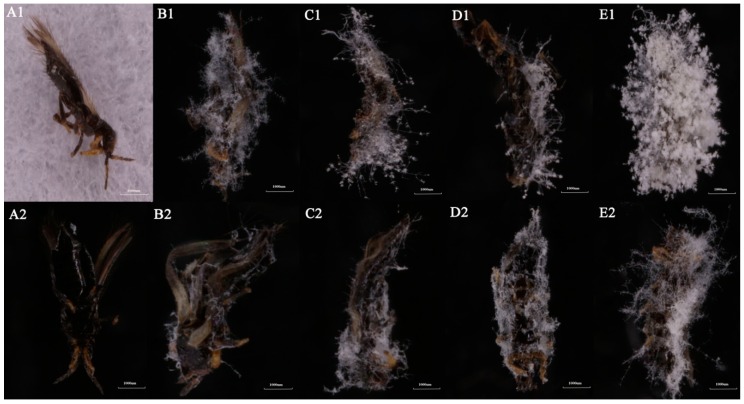
Images of *M. usitatus* infected with strains SCAUDCL-38 and SCAUDCL-56, observed through a dissecting microscope. (**A1**,**B1**,**C1**,**D1**,**E1**) are SCAUDCL-38-infected *M. usitatus* at 24 h, 48 h, 72h, 96 h, and 120 h post-inoculation; (**A2**,**B2**,**C2**,**D2**,**E2**) are SCAUDCL-56-infected *M. usitatus* at 24 h, 48 h, 72 h, 96 h, and 120 eh post-inoculation.

Both SCAUDLC-38 and SCAUDCL-56 induced similar symptoms in adult *M. usitatus*. At 24 h post-inoculation, white hyphae were produced by fungi around the anus and genitals of *M. usitatus* (Figure 4A1,A2). At 48 h post-inoculation, white hyphae covered the whole insect body; however, more white hyphae developed over the head, dorsal trunk, and ventral side of the wings (Figure 4B1,B2). At 72 h and 96 h post-inoculation, the insect behavior was abnormal, and hyphal growth extended over the whole body (Figure 4C1,C2,D1,D2). After 120 h, dense white hyphae completely covered the insects’ body (Figure 4E1,E2).

**Figure 5:**
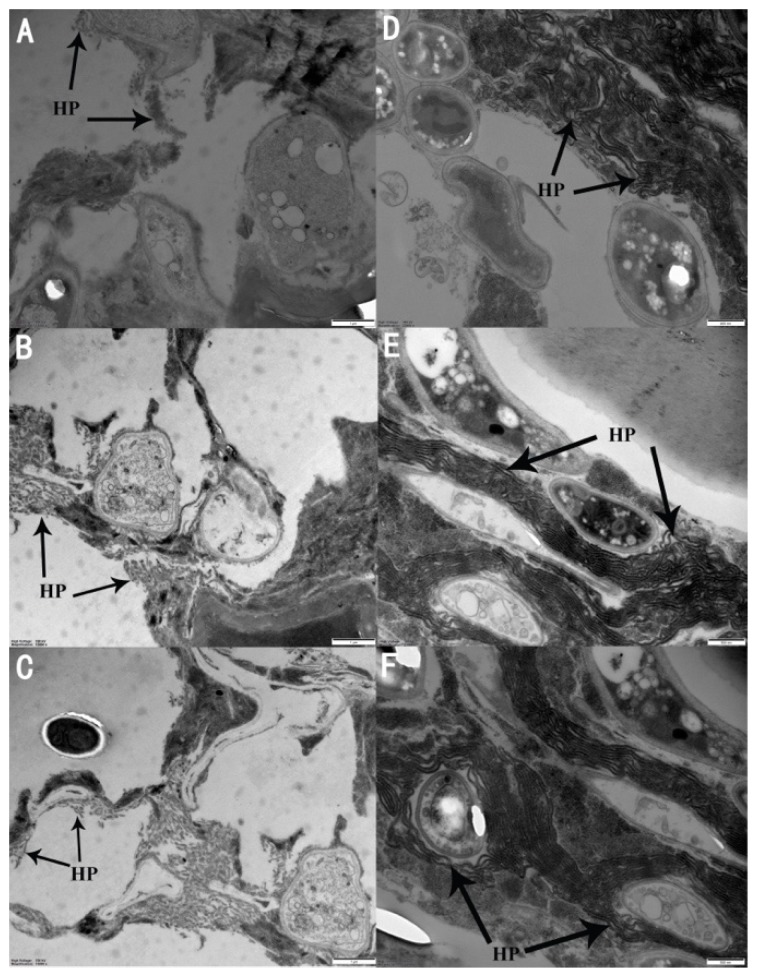
Scanning electron microscope images of the cross sections of *M. usitatus* bodies showing the growth of *A. attenuatus* in *M. usitatus*. (**A**–**C**) Five days post-infection with SCAUDCL-38; (**D**–**F**) five days post-infection infection with SCAUDCL-56. HP: hyphae.

Scanning electron microscope images clearly showed the development of fungal hyphae throughout the body of *M. usitatus* (Figure 5). The results indicated that the adults were infected and killed by *A. attenuatus*.

## 4. Discussion

The development of biological control agents as an alternative to synthetic chemicals requires a clear understanding of the identification and pest control potential of biological control agents. In this study, two isolates of the entomopathogenic fungus *A. attenuatus* were identified and tested for their pathogenicity against *M. usitatus*. Our results revealed the successful isolation and purification of two *A. attenuatus* (SCAUDCL-38 and SCAUDCL-56) isolates from soil samples (collected at the South China Agricultural University in Guangzhou, China, during 2012). Furthermore, dose-dependent mortality studies of the *A. attenuatus* isolates (SCAUDCL-38 and SCAUDCL-56) showed considerable pathogenic potential against *M. usitatus*.

The size of the conidia produced by SCAUDCL-38 and SCAUDCL-56 was 1.5–1.7 × 2.6-6.0 μm and 1.3–2.2 × 3.2–8.2 μm, respectively. The size of the conidia is smaller than that observed for *A. attenuatus* strain ZJLA08 (1.5–2.5 × 3.5–7.0 μm) isolated in China by Lu et al. [20]. Such difference in conidial size may be the result of differences in the respective size of an insect host. Our strains were isolated from the soil, whereas strain ZJLA08 was directly isolated from the insect host (*Diaphorina citri* Kuwayama).

In the current phylogeny of hypocrealean entomopathogens, researchers have realized that the morphological features are not sufficient for the classification and identification of this large and complex fungal group [20,35]. Therefore, the genomic characterization of species can be used to determine the phylogenetic status as well as the identification of a species [36]. Molecular tools based on ITS rDNA genes have been used before to differentiate between morphologically similar *Akanthomyces* species [20]. Our results showed that differences in conidial morphology and size, as well as the homogeneity or variability of conidial size in *Akanthomyces lecanii* species complex (that also includes *A. attenuatus*), were highly correlated with the genomic identification results of the fungi of this species. The pairwise comparisons based on ITS rDNA genes indicated that strains SCAUDCL-38 and SCAUDCL-56 could not be distinguished from *A. attenuatus* (GenBank EF192939).

The ITS sequence has a rapid evolution rate, showing extremely wide sequence polymorphism, and is a highly conserved gene; therefore, ITS sequences are often used for intraspecies and subspecies classification and identification. [23,37]. However, this gene is not sufficient to clearly classify and identify species within the genus *Akanthomyces*, and the classification status of SCAUDCL-56 was not clear in the identification analysis of *Akanthomyces*. In this situation, other genes are usually chosen for sequence analysis of multiple gene loci, and the DNA sequence of the *EF* 1-α gene can successfully distinguish between species of *Akanthomyces* [38]. In this study, the *EF* 1-α sequences classified the Chinese isolates as *A. attenuatus*. Therefore, the phylogenetic tree also confirmed that the isolates SCAUDCL-38 and SCAUDCL-56 belong to *A. attenuatus*.

In the laboratory, *A. attenuatus* SCAUDCL-38 and SCAUDCL-56 both readily produced large quantities of conidia. This is an important reference for future large-scale production of innundative sprays. Our results successfully demonstrated that *M. usitatus* was suceptible to both *A. attenuatus* isolates (SCAUDCL-38 and SCAUDCL-56) in the study. The LC_50_ values of the *A. attenuatus* isolates used were a little higher than those observed by Montalva et al. [39]. These authors studied the toxicity of three strains of *A. attenuatus* (ARSEF13278, ARSEF13279, ARSEF13280) against *Cinara cupressi* (Buckton, 1881); they obtained LC_50_ values of 1.0 × 10^6^, 0.3 × 10^6^, 0.6 × 10^6^ conidia/mL. Kim et al. [40] conducted a virulence test of *A. attenuatus* against *Aphis gossypii* (Glover, 1877), which generated an LT_50_ value of 2.7 days for conidial concentration of 1 × 10^8^ conidia/mL. Our research showed that the highest concentration (1 × 10^8^ conidia/mL) produced LT_50_ values of 3.5 and 4.9 days for SCAUDCL-38 and SCAUDCL-56, respectively. The results of our research differ from those of previous studies, in part because of a different insect host [41,42]. On the basis of these initial research results, we believe that the isolates SCAUDCL-38 and SCAUDCL-56 may be useful candidates for the biological control of *M. usitatus.*

## 5. Conclusions

In summary, the newly identified strains of *A. attenuatus* (SCAUDCL-38 and SCAUDCL-56) were pathogenic to *M. usitatus* under laboratory conditions, having LC_50_ values of 1.9 × 10^6^ and 1.5 × 10^7^ conidia/mL, respectively after five days of fungal treatment. These strains may serve as alternative pest control agents for *M. usitatus*. Further studies are still required to confirm their efficacy under field conditions and to develop optimal formulations.

## Figures and Tables

**Table 1:** Primers used in this study. ITS: internal transcribed spacer, *EF* 1-α: elongation factor 1 alpha.

Sr No.	Genes	Primer	Primer Sequence	Reference
1	ITS	ITS4F	TCCTCCGCTTATTGATATGC	White et al., 1990 [26]
		ITS5R	GGAAGTAAAAGTCGTAACAAGG	White et al., 1990 [26]
2	*EF* 1-α	983F	GCYCCYGGHCAYGGTGAYTTYAT	Rehner and Buckley, 2005 [27]
		2218R	ATGACACCRACRGCRACRGTYTG	Bischoff et al., 2009 [28]

**Table 2:** Reference entomopathogenic fungi from GeneBank used for the phylogenetic analysis.

Species	ITS				*EF* 1-α			
	Accession Number	Strain No.	Host	Location	Accession Number	Strain No.	Host	Location
*A. attenuatum*	EF192939	CNU-23	Green peach aphid	Korea	KM283804	KACC 42493	/	Korea
*A. attenuatum*	MH558279	MO315369	Leaf Roller	USA	EF468782	CBS 402.78	/	USA
*Akanthomyces muscarius*	MH858370	CBS 641.63	/	Albania	KM283821	CBS 143.62	*Trialeurodes vaporariorum*	Korea
*Akanthomyces longisporum*	AJ292385	IMI 021167	Verticillium	United Kingdom	KM283819	CBS 102072	*T. vaporariorum*	Korea
*Akanthomyces antillanum*	AJ292392	CBS 350.85	Verticillium	United Kingdom	DQ522350	CBS 350.85	Animal pathogen	USA
*Akanthomyces dimorphum*	AJ292429	CBS 363.86	Verticillium	United Kingdom	LT220795	TMSL132	Soils	Portugal
*Cordyceps javanica*	JQ425659	BCC24976	Spider	Thailand	KY587206	CHE-CNRCB 357	*Diaphorina citri*	Mexico
*Cordyceps javanica*	JQ425660	BCC26304	Spider	Thailand	KY587208	CHE-CNRCB 363	*D. citri*	Mexico

**Table 3:** Median lethal concentration (LC_50_) values for *A. attenuatus* isolates SCAUDCL-38 and SCAUDCL-56 against *M. usitatus* after five days of fungal treatment.

Isolates	Regression Equation	LC_50_ (Conidia/mL)	95% Confidence Limit
SCAUDCL-38	Y = 0.352X–2.217	1.9 × 10^6^	(3.2 × 10^5^–1.8 × 10^7^)
SCAUDCL-56	Y = 0.229X–1.651	1.5 × 10^7^	(1.2 × 10^6^–9.5 × 10^11^)

**Table 4:** Median lethal time (LT_50_) values for 1 × 10^8^ conidal/mL of *A. attenuatus* isolates SCAUDCL-38 and SCAUDCL-56 against *M. usitatus*.

Isolates	Regression Equation	LT_50_ (Days)	95% Confidence Limit
SCAUDCL-38	Y = 2.901X–5.910	3.52	(2.84–4.75)
SCAUDCL-56	Y = 2.832X–5.864	4.90	(3.82–8.64)
